# Braving the Element: Pancreatic β-Cell Dysfunction and Adaptation in Response to Arsenic Exposure

**DOI:** 10.3389/fendo.2019.00344

**Published:** 2019-06-14

**Authors:** Christopher M. Carmean, Susumu Seino

**Affiliations:** ^1^Division of Molecular and Metabolic Medicine, Department of Physiology and Cell Biology, Kobe University Graduate School of Medicine, Kobe, Japan; ^2^Division of Endocrinology, Diabetes, and Metabolism, Department of Medicine, University of Illinois at Chicago, Chicago, IL, United States

**Keywords:** arsenic, diabetes, pancreas, β-cells, reactive oxygen species, glucose tolerance, endocrine disrupters, insulin secretion

## Abstract

Type 2 diabetes mellitus (T2DM) is a serious global health problem, currently affecting an estimated 451 million people worldwide. T2DM is characterized by hyperglycemia and low insulin relative to the metabolic demand. The precise contributing factors for a given individual vary, but generally include a combination of insulin resistance and insufficient insulin secretion. Ultimately, the progression to diabetes occurs only after β-cells fail to meet the needs of the individual. The stresses placed upon β-cells in this context manifest as increased oxidative damage, local inflammation, and ER stress, often inciting a destructive spiral of β-cell death, increased metabolic stress due to further insufficiency, and additional β-cell death. Several pathways controlling insulin resistance and β-cell adaptation/survival are affected by a class of exogenous bioactive compounds deemed endocrine disrupting chemicals (EDCs). Epidemiological studies have shown that, in several regions throughout the world, exposure to the EDC inorganic arsenic (iAs) correlates significantly with T2DM. It has been proposed that a lifetime of exposure to iAs may exacerbate problems with both insulin sensitivity as well as β-cell function/survival, promoting the development of T2DM. This review focuses on the mechanisms of iAs action as they relate to known adaptive and maladaptive pathways in pancreatic β-cells.

## Introduction

An estimated 451 million people worldwide have type 2 diabetes (T2DM), with as many as 693 million expected to be affected by the disease in 2045 (1). T2DM is characterized by insufficient insulin production relative to metabolic demand resulting in poor glycemic control. In normal glucose homeostasis, a postprandial increase in circulating glucose concentration initiates a spike in insulin secretion from pancreatic β-cells (2, 3). This circulating insulin then binds to its cognate receptor on muscle, liver, and adipose tissues (among others), inducing glucose uptake to lower the concentration of glucose in the bloodstream. In many cases of T2DM, muscle and liver cells (the major sites of glucose disposal in the body) become insulin resistant, which induces β-cells to compensate by secreting more insulin. As insulin resistance becomes more severe, greater stresses are placed on the β-cells to increase their insulin output. Years of this chronic stress on β-cells eventually causes β-cell dysfunction and/or death. With fewer functional β-cells secreting insulin in the context of severe insulin resistance, an inability to properly maintain glucose homeostasis ensues, manifesting as T2DM (4).

Though there are many factors that contribute to the progression of diabetes, it is important to recognize that ultimately a failure of β-cells to secrete sufficient amounts of insulin is what results in hyperglycemia and the diagnosis of T2DM (5). While tremendous stress may be placed on β-cells from a metabolic standpoint, they also face other insults from environmental factors such as endocrine-disrupting chemicals (EDCs). EDCs may work alone or synergistically to derange the normal compensatory mechanisms enabling β-cells to promote glucose tolerance in insulin-resistant individuals (6). In this sense, EDCs may act as drivers of diabetes risk, becoming more deleterious as they compound with lifestyle factors and genetic susceptibilities. It is therefore especially important to consider that EDCs may impair the functionality of β-cells or increase their susceptibility to the chronic metabolic stressors associated with insulin resistance. The purpose of this review will be to explore the β-cell-specific effects of one such EDC, arsenic, as it poses a substantial and ongoing threat to public health.

Arsenic is widely recognized as a carcinogen and oxidizing agent that damages neuronal, hepatic, cardiovascular, integumentary, and renal organ systems (7). Its health effects vary depending on valence, mixture with other toxins, dosage, route of exposure, and length of exposure. Capable of causing death within 24 h, the acute lethal dose in humans is estimated to be 0.6 mg/kg/day (8). Common routes of exposure include the skin, lungs, and digestive tract. Though inhalation of iAs is a concern during hazardous occupational work and traditional coal-based food preservation practices (9), the greatest number of individuals are at risk of exposure to unsafe levels of iAs from contaminated groundwater (10–12). The most prevalent species of arsenic found naturally in drinking water are inorganic arsenic (iAs) in its trivalent (As^III^) or pentavalent (As^V^) forms (13). Organic arsenicals can be found in the food chain as arsenobetaine, arsenocholine, and arsenolipids, and are generally considered relatively non-toxic (14).

Inorganic arsenic is estimated to naturally contaminate the shallow groundwater underneath 140 million people worldwide (12). Among these people at risk, the number actually exposed to iAs is believed to be lower, as not all contaminated groundwater sources are utilized and excellent remediation methods are available in developed countries (12). Despite this fact, the problem of chronic iAs exposure through shallow groundwater consumption persists on an immense scale. As of 2007, an estimated 20 million people in Bangladesh alone were still served by wells naturally contaminated with iAs at a concentration more than 5x higher than the WHO safe limit of 10 μg/L (15). In these areas, where exposure has been pervasive in communities since the digging of shallow groundwater wells in the mid-1900s, it has been estimated that ~21% of all-cause mortality is attributable to iAs exposure (16). The lasting effects of this contamination may represent one of the greatest failures of public health management in recent history.

One observation from this unintended mass-poisoning and other cases of natural exposure is a potential relationship between iAs exposure and diabetes (11). In addition to its role in increasing the risk of several cancers, peripheral neuropathy, and keratinosis, iAs exposure correlates with glucose intolerance or diabetes prevalence in areas with relatively high exposure levels (17–20). The epidemiological analysis regarding the relationship between iAs exposure and diabetes have been reviewed in detail elsewhere (11). For the purposes of this review, an examination of the specific effects of iAs on β-cells will be undertaken, including the evidence from pancreatic endpoints in animal models and mechanistic insights gained from *ex vivo* and/or interventional studies.

## Acute vs. Chronic iAs Exposure

The effects of iAs can be conceptualized on a sliding scale of dosage and time. A single large dose of iAs can cause nausea, vomiting, abdominal pain, diarrhea, and even death (8, 21). Chronic ingestion of lower doses of iAs can occur without any immediate sensory feedback, and yet the ensuing damage over several years may span most organ systems, increasing an individual's risk of cancer, peripheral neuropathy, cardiovascular disease, diabetes, and a multitude of skin problems (7). In trying to better understand the relationship between iAs exposure and diabetes, the effects of chronic, sub-toxic exposure are of the greatest concern and should be delineated from acute toxic effects.

The National Toxicology Program Workshop Review's assessment in 2012 suggested that cell-culture studies utilizing iAs concentrations ≥ 1 mM can be considered acute stress-response studies rather than functional studies of β-cells' role in iAs-associated diabetes, even in cases where physiologically-relevant model systems or physiological endpoints were utilized (11). Given that the highest circulating plasma concentration of iAs ever recorded in a human population drinking iAs-contaminated water was 0.6 μM, and clear evidence of reduced cell growth or survival *ex vivo* has been reported for iAs exposures ≥5 μM (22, 23), 1 mM appears to be a generous and appropriate cutoff (24, 25).

Although studies have repeatedly shown that exposure to ≤1 μM iAs significantly decreases glucose-induced insulin secretion in clonal β-cells, it should be noted that the use of somewhat higher concentrations for short periods has spurned fruitful follow-up at lower, more physiological concentrations of iAs. For instance, Pi et al reported that 5 μM iAs significantly induced antioxidant gene expression (26), eventually leading to deeper investigation of the role of nuclear factor (erythroid-derived)-like 2 (Nrf2), a major antioxidant-regulating transcription factor, in the adaptive response to iAs. This launched a series of investigations that expanded our understanding of iAs's mechanism(s) of action (27, 28). Given the fine line between the concentration of iAs capable of inhibiting insulin secretion and cytotoxicity or the induction of apoptosis, this course of studies might be taken as an excellent example of how to successfully exploit shorter time courses and higher dosages to generate novel, environmentally-relevant findings.

## Pancreatic iAs Accumulation, Speciation, Disposal

IAs undergoes several stages of metabolism once ingested (29). IAs enters cells via ion transporters, including aquaporin proteins 3, 7, and 9 and glucose transporter 1 (30–33). Although a reduction of these transporters protects cell lines against iAs toxicity (34), efficient cellular import of arsenicals is critical for normal *in vivo* iAs detoxification and urinary excretion (35). A single sub-lethal bolus of iAs (1 mg/kg) administered intraperitoneally to rats, mice, hamsters, and guinea pigs, can be cleared from the bloodstream in ~24 h (36). Once inside the cell, iAs may be conjugated to glutathione or methylated multiple times (37, 38). Once modified, methylated or glutathione-conjugated arsenicals are exported from the cell by ABC transporters, including multidrug resistance-associated proteins 1a, 1b, and 2 (39, 40), enabling more efficient renal excretion and minimizing internal exposure. Arsenical efflux activity partially determines an organism's sensitivity to iAs, as the activities and expression levels of these ABC transporters are critical for adaptation to iAs (31).

IAs is taken up by pancreatic tissue and β-cells dose-dependently (41). Of the *in vivo* rodent studies utilizing chronic iAs exposure considered here, several studies specifically measured iAs accumulation in the pancreas (Table 1). In all these cases except one, iAs accumulated significantly in the pancreas (41, 43, 44, 46, 57–60, 64, 74). In the one exception, with low levels of iAs used in a cocktail of other dilute toxins, no appreciable pancreatic iAs accumulation was observed (77). The concentration of iAs used in this study by Radike et al was lower than the World Health Organization's safe limit of 10 μg/L.

**Table 1:** Rodent models of iAs exposure with pancreatic endpoints.

**Animal model**	**As species**	**Dose (route)**	**Time**	**Pancreatic As Accumulation**	**Glucose tolerance effects**	**Insulin effects**	**Pancreatic endpoints**	**General findings**	**References**
Wistar rat (m)	As_2_O_3_	17.75–100 mg/L (dw)	8 wk	NR	OGTT glucose clearance delayed OGTT AUC lower	Fasting blood insulin ϕ	NR	Oxidative stress induces mito dysfunction	(42)
Wistar rat (m)	As^III^	1.7 mg/kg(og)	2x daily 90 d	Yes	Fasting glucose ↑ HOMA-IR ↑	Fasting insulin ↑ Glucose:insulin ratio ↓	Glucagon staining ↓ Insulin IHC signal ↓	Serum glucagon ↓	(43)
C57BL/6 mouse (m)	As^III^	25–50 ppm(og)	20 wk	Yes	FBG in HFD + iAs group was lower than HFD controls. OGTT AUC for iAs exposed mice in high fat diet group ϕ HOMA-IR in HFD + iAs group was lower than HFD controls	Insulin during first 15 min of OGTT in high-fat diet group iAs exposed mice ↓	NR	Water intake ↓ HFD iAs adiposity was lower than HFD controls	(44)
LM/Bc/Fnn mouse (gf)	Na_2_HAsO_4_	9.6 mg/kg(ip)	2 doses (G7.5 and G8.5)	NR	FPG ↑ Glucose tolerance ↓ (IPGTT) RPG ↑	Fasting insulin ϕ IPGTT insulin at 30 min ↑	NR	NAC, methionine, sodium selenate,	(45)
ICR mouse (m)	As_2_O_3_	5 ppm(dw)	6 wk	Yes	FPG ϕ Glucose tolerance (ogtt) ↓	Fasting insulin ↓	Markers of β-cell apoptosis ↑	NAC co-improved glucose tolerance	(46)
Sprague-Dawley rat (mf)	Mix As + Pb^*^	30 ppb As, 53 ppb Pb(dw)	3 mo	NR	f rat FPG ↓ m insulin resistance ↑ f insulin sensitivity ↑ mf glucose intolerant (OGTT)	f OGTT insulin ϕ m OGTT insulin ↑	NR	NR	(47)
C57/BLKS/J db/m and C57BKS/Lepr^db^ (db/db)mouse (m)	As^III^	3 mg/L (dw)	16 wk	NR	HOMA-IR ϕ Normal mice glucose tolerance ϕ (OGTT) Db/db mice glucose tolerance ↓	FBI ↑ db/db mice HOMA-β ↓ db/db mice FBI ↓	Normal mice HOMA-β ↑ As worsened inflammation	Daily food intake altered Daily water intake ϕ Hepatic gluconeogenesis ↑	(48)
Sprague Dawley rat (gf, o)	As^III^	5–50 mg/L(dw)	8 wk + gestation from day 1	NR	Gestational BG IPGTT ↓ HOMA-IR ϕ Gestational FPG ϕ	Gestational circulating insulin IPGTT ↓m and f offspring insulin AUC IPGTT ↓	Gestational panc insulin ↑	Daily water consumption ϕMaternal weight gain ↓	(49)
Sprague Dawley rat (mf)	As^III^	5–50 mg/L(o)	8 wk + gestation from day 1	NR	mf FPG ϕ f BG IPGTT ↓ f HOMA-IR ↑ m HOMA-IR ↓ m BG IPGTT ϕ	Mf insulin IPGTT ↓	NR	Body weight ↓ Hepatic GSH ↑ Hepatic MDA ↑	(49)
C57BL/6J mouse (m)	As^III^	50 mg/L(dw)	8 wk	NR	Glucose tolerance (IPGTT) ↓ HOMA-IR ↓	IPGTT 1st phase Insulin vs. glucose ↓	Pancreas mass ↓	Water intake ↓ Circadian feeding pattern disrupted	(50)
C57BL/6J mouse (m)	As_2_O_3_	1–4 mg/L(dw)	12 wk	NR	NR	Harvested islet GIIS ↓	ER stress ↑ Autophagy ↑	NR	(51)
NMRI mouse (m)	As^III^	25–50 ppm(dw)	20 wk	NR	HFD-fed mice + As FPG ↓ HFD-fed mice + As HFD + As HOMA-IR ↓	HFD + As FPI ↓	Pancreas mass ↓ HFD + As islet diameter ↓	Water consumption ↓	(52)
					Glucose tolerance in HFD + As group vs. HFD only (OGTT) ↓				
C57BL/6J mouse (mf)	As^III^	100–1000 ppb(dw)	1 wk before and 1st wk of pregnancy	NR	Male prenatal As exposure adulthood FPG ↑ Male prenatal As exposure adulthood HOMA-IR ϕ	FPI ϕ	NR	Male prenatal 1 ppm body fat % ↑ HOMA-IR in males at 100 ppb ↑	(53)
Albino rat (m)	As_2_O_3_	3 mg/kg(og)	Daily, 30 days	NR	NR	NR	Islet size ↓ Markers of ROS ↑ NO ↑	Folic acid intervention	(54)
Albino Wistar rat (m)	As^III^	1.5 or 5 mg/kg (og)	5 wk	NR	≥ 1.5 mg/kg FBG ↑ ≥ 1.5 mg/kg HbA1C ↑ OGTT glucose ↑	NR	Antioxidant activities ↑ Oxidative stress ↑	Zn and Cu ↓	(55)
Wistar rat (gf, o)	As_2_O_3_	2–8 mg/kg(og)	G6 to postnatal day 42	NR	NR	NR	Islet size ↓ autophagosomes ↑ LC3-II ↑ Nrf2, Trx ↓	Taurine intervention	(56)
C57BL/6 mouse (m)	As^III^	10, 25, 50 ppm(dw)	8 wk	Yes	FPG ϕ 50 ppm OGTT blood glucose ↑	NR	NR	NR	(57)
C57BL/6 mouse (m)	MMA	2.5, 5 ppm(dw)	8 wk	Yes	FPG ϕ OGTT blood glucose ϕ	NR	NR	NR	(57)
C57BL/6 mouse (mf)	As^III^	10 mg/kg(ip)	Bolus	NR	Fasting glucose ↓	NR	NR	Some mice died after 1 day	(21)
Wistar rat (m)	Diphenylarsinic acid (DPAA)	5 mg As/kg (og)	Bolus	Yes	NR	NR	NR	Highest DPAA accumulation in brain	(58)
Swiss albino mouse (NR)	As^III^	3 mg/kg(og)	Daily for 12 wk	Yes	FPG ↑	NR	NR	(6)-gingerol intervention	(59)
Sprague Dawley rat (m)	NaAsO_2_	0.5–10 ppm(dw)	8 wk	Yes	NR	NR	Pancreas mass ↓	As accumulated in every organ examined	(41)
C57BL/6J mouse (m)	As^III^	15–50 mg/L(dw)	4 wk	Yes	NR	NR	NR	As3mt-KO mice + As water intake ↓	(60)
CD rat (m)	As^III^	5–10 mg/kg(ip)	Bolus or daily for 7 d	NR	Single dose or 7d iAs, fasting blood glucose ↑ Single dose or 7d iAs, OGTT blood glucose ↑	NR	NR	Adrenalectomy partially prevented glucose intolerance after iAs	(61)
SD rat (m)	As^III^	0.1–1 mg/kg(ip)	Bolus	NR	FBG ϕ	NR	NR	Kidney PDH activity ↓	(36)
B6C3F1 mouse (m)	As^III^	0.1–1 mg/kg(ip)	Bolus	NR	FBG ϕ	NR	NR	Kidney PDH activity ↓	(36)
Golden-Syrian hamster (m)	As^III^	0.1–1 mg/kg(ip)	Bolus	NR	FBG ϕ	NR	NR	Kidney PDH activity ↓	(36)
Hartley guinea pig (m)	As^III^	1 mg/kg(ip)	Bolus	NR	FBG ↑	NR	NR	Kidney PDH activity ↓	(36)
Wistar rat (m)	As^III^	5.55 mg/kg(ip)	Daily for 21 d	NR	FPG ↓ (rats were hypogycemic)	NR	NR	Liver glycogen ↓Methionine intervention	(62)
Wistar rat (m)	As^III^	5.55 mg/kg(ip)	Daily for 30 d	NR	FPG ↓ (rats were hypogycemic)	NR	NR	Oral NAC intervention	(63)
C57BL/6 mouse (m)	As^III^	25–50 ppm(dw)	8 wk	Yes	FPG ϕ IPGTT glucose ↑	NR	NR	Water consumption ↓	(64)
Wistar rat (m)	As^III^	5 mg/kg(og)	Daily for 30 d	NR	FPG ↑	NR	NR	Curcumin intervention	(65)
ICR mouse (f)	As_2_O_3_	0.05–0.5 mg/kg(dw)	2–6 wk	NR	As only group became glucose intolerant Ovariectomized + As had worst Ovariectomized + As + estrogen restored glucose tolerance	0.05, 0.5 μM AS, 2, 4, 6 wk FPI ↓ 2 4, 6, wk, 0.05, 0.5 μM AS, ovariectomized mice + iAs ↑ 2 4, 6, wk ovariectomized mice + iAs + estradiol ϕ	NR	Liver glycogen ↓ Body fat % ϕ Estradiol intervention	(66)
Sprague-Dawley rat (m)	As^III^	8 mg/kg (ip)	1 dose	NR	IPGTT BG ↑	NR	NR	NAC improved glucose tolerance	(67)
Sprague-Dawley rat (m)	As^III^	20–200 ppm(dw)	20 wk	NR	FBG ϕ IPGTT BG ϕ	NR	NR	NR	(67)
CD-1 ICR mouse (m)	As_2_O_3_	10 mg/L (dw)	3–12 wk	NR	NR	5–12 wk FPI ↓	5 wk inflammatory cells ↑ and acinar cells ↓	Humic acid also decreases FPI	(68)
B6C3F1 mouse (mf)	MMA	10–400 ppm (food)	2 yr	NR	FBG ϕ	NR	Adenoma/carcinoma ϕ	Water consumption ↑	(69)
Fischer F344 rat (mf)	MMA	50–1,300 ppm (food)	2 yr	NR	FBG ϕ	NR	Adenoma ↑	Water consumption ↑	(69)
Wistar rat (mf)	As_2_O_3_	2–8 mg/kg (og)	Daily for 56 d	NR	NR	NR	ROS ↑ Mitophagy ↓	Taurine restored mitophagy	(70)
Wistar rat (mf)	As_2_O_3_	2–8 mg/kg (og) (gf)	Daily post-weaning for 14 d	NR	NR	NR	Irregular structures Inflammasome ↑	Taurine restored structure and reduced inflammation	(71)
CD1 mouse (m)	As^III^	20–40 ppm (dw)	52 wk (dw)	NR	OGTT glucose AUC ↑	Fasting insulin ↓ OGTT insulin fold-change ↓ Islet GIIS ↓ Islet Insulin content ↓	mafA mRNA ↓ mir-149, mir-153 ↑	NR	(72)
ICR mouse (m)	As^III^	10 ppb	G10–G18	NR	IPGTT ϕ	NR	NR	BPA + iAs IPGTT AUC ↓	(73)
Balb/C mouse (m)	As^III^	5 μM	6 wk (dw)	Yes	FPG ↑	NR	Pancreas morphology ϕ Pancreas miR-2909 ↓	NR	(74)
C57BL/6J mouse	As^III^	100 ppb	6–37 wk (dw)	NR	FBG ϕ Insulin tolerance ϕ	FPI ϕ	NR	Sex-specific enhanced iAs metabolism with folate sufficiency	(75)
NMRI mouse (m)	As^III^	50 ppm	20 wk	NR	HFD FBG ↓ HFD HOMA-IR ↓	NC and HFD FSI ↓ NC and HFD HOMA-β ↓	NR	Liver ROS ↑ Liver lipid peroxidation ↑	(76)

*m, male; f, female; gf, gravid female; ip, intraperitoneal injection; og, oral gavage; dw, drinking water; NR, not reported; d, days; wk, weeks; yr, year; G, gestational day; FBG, fasting blood glucose; FPG, fasting plasma glucose; RPG, random plasma glucose; FSI, fasting plasma insulin; o, offspring exposed to iAs during gestation; OGTT, oral glucose tolerance test; IPGTT, intraperitoneal tolerance test; MMA, monomethylarsenous acid; DMA, dimethylarsenous acid*.

* *Chemical identities of iAs were not described and samples were directly taken from a drinking water source*.

Chronic administration of iAs in drinking water results in iAs accumulation as both iAs and methylated arsenicals in the pancreas, with the majority stored as monomethyl arsenous acid (MMA) or dimethylarsenous acid (DMA) (43, 44, 57, 60, 64). Isolated islet and β-cell cell line studies have demonstrated the ability of β-cells to methylate iAs intracellularly (78, 79). The physiological effects of these methylated arsenicals appear to be different from those of iAs. MMA can inhibit mitochondrial function and decrease glucose-induced insulin secretion, even at 5-fold lower concentrations than other arsenicals (80). Since MMA is necessarily created prior to repeated bouts of methylation resulting in dimethylation and trimethylation, it is noteworthy that this intermediate may be more toxic than its precursor or end-products.

The relationship between exposure and tissue-level accumulation as well as the propensity to induce DNA damage vary across organ systems. One study measured iAs accumulation and cytosine methylation in several tissues following 24 weeks of iAs administration (41). Relative to the lung, kidney, heart, and spleen, the pancreas accumulated less iAs as a function of exposure level and displayed a resistance to the iAs-induced 5-hydroxymethylation events observed in these other tissues. Despite this apparent resistance, the pancreas itself was smaller after adjusting for body weight in iAs-exposed mice, a phenomenon also observed by other groups (50, 52), raising the possibility that the organ may possess a unique resistance to iAs accumulation, but also a unique susceptibility to the effects of iAs exposure.

## Model System Evidence for Pancreatic β-Cell Involvement

The data are mixed in animal models of iAs-induced metabolic dysfunction regarding the relative contributions of insulin-secretory vs. insulin-sensitivity factors in the development of glucose intolerance (Table 1). Impairments in pancreatic (41, 50–52, 54–56, 68, 69) and hepatic (48, 55, 59, 61–63, 66) function have been implicated. Insulin sensitivity, however, has been reported to increase (44), decrease (43, 47, 49, 53), or remain unchanged (44, 47, 50) in sex-specific or diet-specific manners, making the integration of this particular endpoint across studies more difficult. It is worth noting, however, that where insulin sensitivity was reported to increase, this was on a background of already-impaired diet-induced glucose intolerance in which Paul et al also reported both lower circulating insulin, lower adiposity, and lower HOMA-IR in the high-fat diet, iAs-treated group vs. high-fat diet controls. In this study as well as others, a reduction in circulating insulin was reported either following fasting or during a glucose tolerance test (44, 46, 50, 57–60, 64, 74, 77–80). In consideration of the studies examined here, it appears that a primary defect in β-cell function precedes the development of glucose intolerance, which may or may not include a component of insulin resistance. IAs may be protective against insulin resistance in diet-induced obesity while simultaneously impairing pancreatic β-cells, a model that deviates from the canonical type I, type II, or gestational forms of diabetes (81). Replication will be critical for reconciling the differences between insulin sensitivity outcomes in these recent animal models of iAs exposure.

In β-cell lines there is a consistently observed reduction in GIIS associated with chronic, sub-toxic iAs exposure (Table 2), although there is some disagreement in the literature about whether basal insulin secretion is altered by iAs exposure (23, 28, 42, 82, 83). Some of these differences may be related to the model systems employed. It is notable that, in cell line studies, the dosages and times used to study the effects of iAs exposure have varied dramatically. Timeframes utilized for studying the effects of GIIS have ranged from 1 to 144 h, with higher concentrations evaluated on shorter time courses, such as 5 mM iAs exposure for 60 min (22) or over 100 μM for 90 min (86, 87), and lower doses for longer time courses, such as 50 nM for 96 h (28). The lowest concentration thus far reported to significantly affect GIIS in cell culture studies is 0.1 μM (23).

**Table 2:** *Ex vivo* models of iAs exposure.

**Model**	**Tissue type**	**As species**	**Dosage**	**Time**	**Insulin content**	**Insulin secretion**	**General findings**	**References**
Wistar rat (m)	Islets	As_2_O_3_	NR	NR	NR	Basal ↑ GIIS ↓	O_2_ consumption ↓	(42)
rat	INS-1	As^III^	0.05–0.5 μM	96 h	Insulin mRNA ↑ Insulin ↑	Basal ↑ GIIS ↓ KIIS ↑	H_2_O_2_ scavenging ↑ Nrf2-regulated gene mRNA ↑ Mito mass ↑	(28)
C57BL/6J mouse (m)	Islets	As^III^	0.1–2 μM	48 h	0.1–2 μM ϕ	2 μM GIIS ↓ 2 μM KIIS ↓	iAs^III^ accumulated in islets after 48 h exposure	(78)
rat	INS-1	MAs^III^	0.1–2 μM	2–24 h	NR	4+ h, 2 μM GIIS ↓ 24 h, 0.375 μM GIIS ↓	≥ 0.2 μM Mito respiration impaired	(80)
IT6 mouse	MIN6-K8	As^III^	0.1–2 μM	72 h	NR	Basal ϕGIIS ↓	O_2_ consumption ϕ Dependent on intact 5HT metabolism 5-HT disposal ↑ *Ugt1a6a* mRNA ↑ Supplementation with 5HTP recovered GIIS	(23)
C57BL/6J mouse (m)	Islets	As^III^	2 μM	48 h	NR	Basal ϕ GIIS ↓	*Ugt1a6a* mRNA ↑	(23)
human	Islets	As^III^	1–2 μM	72 h	NR	Basal ϕ GIIS ↓	*UGT1A6* mRNA ↑	(23)
rat	RINm5F	As^III^	0.5–5 μM	72 h	0.5–5 μM insulin ϕ	1, 2 μM Basal ↓ 2 μM GIIS ↓	Glucose-induced Ca2+ ↓ Glucose-induced SNAP-25 proteolysis ↓ Cell cycle arrested	(82)
Wistar rat (m)	1°β-cells	As^III^	0.5–10 μM	72–144 h	1 μM ϕ 5 μM ↓ 5 μM insulin mRNA ↓	Basal ϕ 1, 5 μM GIIS ↓	5 μM Insulin mRNA ↓	(83)
rat	RINm5F	As_2_O_3_	0.5–10 μM	4–8 h	NR	2 μM, 5 μM, 24 h GIIS ↓	Calpain activity ↑ ROS ↑ Supplementation with NAC decreased apoptosis	(46)
IT6 mouse	MIN6	As^III^	1–20 μM	2–24 h	NR	NR	iAs accumulates as iAs and MMA Antioxidant gene mRNA ↑	(79)
129S1/SvImj mouse (NR)	Islets	As^III^	1–10 μM	7–15 h	NR	Basal ϕ 1 μM, 15 h GIIS ↓	ROS-scavenging genes and protein ↑	(26)
Hamster	HIT-T15 β-cells	As_2_O_3_	1 μM−25 μM	2–24 h	4 h, ≤ 2 μM ϕ 4 h, 5 μM ↓	NR	≥ 2.5 μM, 2, 8 or 24 h ATP ↓ LD_50_ = 2.5 μM 5–20 μM 2, 4 h ROS ↑	(68)
Rat	INS-1	As^III^	5–50 μM	6–24 h	NR	5 μM, 6 h GIIS ↓	Glucose-induced H_2_O_2_ ↓	(26)
	Islets	As^III^	1 μM	15 h	NR	Basal ϕ GIIS ↓	Basal H_2_O_2_ ↑ Glucose-stimulated H_2_O_2_ ↓	(26)
Rat	INS-1	As^III^	1–10 μM	2–24 h	NR	6 h, 10 μM GIIS ↓ 24 h 2 μM GIIS ↓	2 μM OCR ↓	(80)
Rat	INS-1	As^III^	1–4 μM	24 h	NR	NR	≥ 2 μM Viability ↓ ≥ 2 μM Autophagosomes ↑ ≥1 μM LC3II protein ↑ NAC intervention	(84)
Rat	INS-1	DMAs^III^	2–10 μM	2–24 h	NR	2+ h, 10 μM GIIS ↓ 24 h, ≤ 2 μM GIIS ϕ	≤ 2 μM Mito respiration ϕ	(80)
rat	INS-1	As^III^	2.5 μM−160 μM	12–72 h	NR	NR	≥ 2.5 μM AS is cytotoxic ≥ 2.5 μM AS causes L3/CytC apoptosis	(85)
rat	INS-1	As^III^	4 μM	3–24 h	NR	NR	12, 24 h LC3-II protein ↑ 24 h p62 protein ↓ 6–24 h ER stress ↑	(51)
rat	INS-1	As^III^	5 μM	6–15 h	NR	Basal ϕ GIIS ↓	Nrf2 activation ↑ Antioxidant activity ↑ Antioxidant gene + protein expression ↑ H_2_O_2_ accumulation ↓	(26)
Swiss Albino mouse	1°β-cells	As^III^	10 μM	72 h	NR	NR	72 h, 10 μM ROS ↑	(59)
NMRI mouse (m)	Islets	As^III^	20–200 μM	90 min	NR	≥ 100 μM GIIS ↓	≥ 50 μM viability ↓ ≥ 100 μM ROS ↑	(86)
NMRI mouse (m)	Islets	As^III^	100 μM	90 min	NR	GIIS ↓	Metformin pre-treatment protected GIIS	(87)
Human	Islets	As^III^	1 mM	30 min	NR	NR	Nuclear PDX1 ↑	(88)
Human	Islets	As^III^	1 mM	15–30 min	NR	NR	UIF1 DNA binding ↑	(89)
IT6 mouse	MIN6	As^III^	1 mM	5–20 min	NR	NR	MAPKAP-K2 activity ↑	(89)
Ob/Ob mouse (m)	Islets	As^V^	5 mM	60 min	NR	Basal ϕ GIIS ↓	Glucose-stimulated O_2_ consumption ↓	(22)
Rat	INS-1	As_2_O_3_	1–4 μM	24 h	NR	NR	Apoptosis ↑ PPARγ ↓ Taurine decreased apoptosis	(70)
rat	INS-1	As_2_O_3_	1–64 μM	24 h	NR	4 μM As_2_O_3_ + LPS Basal ↓ 4 μM As_2_O_3_ + LPS GIIS ↓	As_2_O_3_ + LPS Pyroptosis ↑ As_2_O_3_ + LPS GIIS ↓ Taurine partially restored GIIS Inflammasome inhibitors partially restored GIIS ↓	(71)
IT6 mouse	MIN6	As^III^	1–5 μM	6–48 h	≥2 μM ≥24 h insulin content ↓	2 μM ≥12 h GIIS ↓	2 μM 48 h mir-149 ↑ Mir-149 knockdown restored GIIS through mafA Mir-149 knockdown restored insulin content	(72)
IT6 mouse	MIN6	As^III^	2 μM	24 h	NR	2 μM 24 h Basal ϕ	2 μM 24 h miR-2909 ↑ 2 μM 24 h MafA mRNA ↓ 2 μM 24 h MafA protein ↓ 2 μM 24 h PDX1, C-Jun, UCP2 protein ↑	(74)
rat	INS-1 832/13	As^III^	1–2 μM	24 h	NR	≥1 μM 24 h GIIS ↓	≥1 μM 24 h XTT viability ↓ 2 μM 24 h basal and glucose-stimulated OCR ↓ ≥1 μM 24 h maximal OCR ↓	(90)

*NR, not reported; m, male; f, female; MMA, monomethylarsenous acid; DMA, dimethylarsenous acid; GIIS, glucose-induced insulin secretion; KIIS, potassium-induced insulin secretion; OCR, oxygen consumption rate*.

Significant effects of iAs on insulin gene expression and transcription factor activities have also been reported. A decrease of MafA transcriptional activity regulated by miR-149 may contribute to the iAs-induced impairment of β-cell function (72). Such decreases in *MafA, Pdx1*, or *Nkx6.1* are generally considered indications of β-cell failure or de-differentiation (91). Other model systems of iAs exposure, in which gene expression levels of these transcription factors were measured, did not report such a de-differentiation phenotype (23). One study even observed an increase in nuclear PDX1 following exposure to iAs, suggestive of increased insulin gene expression (88). DNA binding of the β-cell specific transcription factor UIF1, which promotes insulin gene expression, has also been observed to increase in response to iAs, suggesting a mechanism by which iAs may affect insulin content (89). Additional replication may therefore be warranted to identify which features of transcription factor activities are robust and translatable to human exposure.

## Inflammation and Reactive-Oxygen Species (ROS)

ROS accumulation is a hallmark of iAs toxicity. There is strong evidence from both *in vivo* and *in vitro* studies suggesting that iAs damages pancreatic tissue, observed as elevated pro-inflammatory genes (48), pancreatic nitric oxide (54, 55) glutathione levels (43, 55), endoplasmic reticulum stress (51), and autophagy (51, 56). More severe phenotypes have been observed with increased apoptosis (46), decreased islet size (54), accumulation of pro-inflammatory cells (68), and detection of pancreatic adenomas (69). The generalized cellular responses to iAs-induced ROS have been reviewed in detail elsewhere (92). Markers of ROS have been observed at the lowest concentrations that also affect GIIS (28). Interestingly, arsenic trioxide (As_2_O_3_) appears to increase ROS production, apoptosis, and TUNEL staining while decreasing PPARγ in the INS-1 cell line. Restoration of normal ROS production by taurine administration or rescue of PPARγ expression ameliorates the apoptotic and DNA-damaging effects of As_2_O_3_ exposure (70). This is in line with similar observations for liver cells lines by the same group (93).

The ROS produced as a result of iAs exposure induce a compensatory increase in gene expression levels for genes regulated by antioxidant response elements (26–28, 94). These genes, which include catalase, superoxide dismutase 1, and superoxide dismutase 2, are critical for reducing otherwise toxic accumulation of ROS, and are positively regulated at the level of transcription by Nrf2 (94, 95). In β-cells specifically, induction of the Nrf2-mediated antioxidant-response program has been shown to protect against iAs-induced toxicity (94). Deletion of the major transcription factor regulating this pathway, Nrf2, in β-cells has been shown to enhance susceptibility to iAs toxicity (79). In the context of β-cell function, this antioxidant activity may actually suppress the normal physiological changes in ROS that β-cells depend on to induce insulin secretion in response to a rise in extracellular glucose (26). In this way, a tradeoff may occur in which β-cells' survival improves by adaptive upregulation of antioxidant activity, while at the same time glucose-induce insulin secretion is suppressed by the same mechanism (96). That several interventional studies focused on suppressing the antioxidant response successfully ameliorated some of the effects of iAs supports the hypothesis that ROS may be one of the salient, translatable, and addressable features of low-dose, chronic iAs exposure (Table 3) (45, 46, 53, 59, 63, 65, 67, 71, 75, 84, 97).

**Table 3:** *In vivo* interventional studies.

**Biological and Exposure Model**	**Intervention (substance/dose)**	**Effects/proposed mechanism**	**References**
Wistar rats, 5.55 mg/kg/day As^III^ (ip) for 30 days	N-acetylcysteine, 1 mmol/kg/day (og)for final 7 days of As^III^ exposure	Nacetylcysteine's anti-oxidant properties reversed iAs-induced hepatic ROS-mediated toxicity, restored lower liver glycogen levels, and reversed hypoglycemia	(63)
Wistar rats, 5.55 mg/kg/day As^III^ (ip) for 30 days	Melatonin, 10 mg/kg/day (og) for final 5 days of As^III^ exposure	Melatonin's anti-oxidant properties reversed iAs-induced reductions in superoxide dismutase and catalase activities in the liver and kidney.	(97)
Wistar rats, 5.55 mg/kg/day As^III^ (ip) for 21 days	Methionine, 0.8% of food supplement for final 5 days of As^III^ exposure	Methionine treatment may have enhanced methylation of iAs reduced its toxicity, reversed hypoglycemia, reversed the iAs-induced reduction in liver pyruvic acid, and partially reversed the reduction in liver glycogen levels.	(62)
Swiss-albino mice, 3 mg/kg/day As^III^ (og) for 12 weeks	(6)-gingerol, 50–75 mg/kg body weight/day (og) for 3 weeks after As^III^ exposure	(6)-gingerol administration restored iAs-induced hyperglycemia to normoglycemia, decreased iAs deposition in the pancreas and liver, and restored liver antioxidant activities.	(59)
Wistar rats, 8 mg/kg/day As_2_O_3_ (og) from GD 6 to postnatal day 42	Taurine, 150 mg/kg/day (og) from GD 6 to postnatal day 42	Taurine reversed iAs-induced autophagosome formation, iAs-induced decrease in Nrf2 protein levels, and iAs-induced ROS accumulation in the pancreas.	(56)
Wistar rats, 8 mg/kg/day As_2_O_3_ (og) from GD 6 to postnatal day 42	Taurine, 150 mg/kg/day (og) from GD 6 to postnatal day 42	Taurine reversed iAs-induced TNF-α expression and markers of pyroptosis and inflammation in the pancreas.	(71)
Pregnant LM/Bc/Fnn mice, 9.6 mg/kg As^III^ (ip), at GD 7.5 and GD 8.5	Sodium selenate, 0.5 mg/kg (og) daily from GD 0.5 to GD 10.5	Sodium selenite decreased the number of fetuses with neural tube defects.	(45)
Pregnant LM/Bc/Fnn mice, 9.6 mg/kg As^III^ (ip), at GD 7.5 and GD 8.5	L-Methionine, 70 mg/kg (og) daily from GD 0.5 to GD 10.5	L-Methionine decreased the number of fetuses with neural tube defects.	(45)
Pregnant LM/Bc/Fnn mice, 9.6 mg/kg As^III^ (ip), at GD 7.5 and GD 8.5	N-acetylsysteine, 200 mg/kg (og) daily from GD 0.5 to GD 10.5	N-acetylsysteine decreased the number of fetuses with neural tube defects but did not affect FPG or maternal circulating insulin	(45)
Pregnant LM/Bc/Fnn mice, 9.6 mg/kg As^III^ (ip), at GD 7.5 and GD 8.5	N-tert-Butyl-α-phenylnitrone,40 mg/kg (ip) on GD 7.5 and GD 8.5	N-tert-Butyl-α-phenylnitrone decreased the number of fetuses with neural tube defects and significantly increased the rate of fetal resorption	(45)
Pregnant LM/Bc/Fnn mice, 9.6 mg/kg As^III^ (ip), at GD 7.5 and GD 8.5	LinBit insulin pellet implanted from GD 2.5–3.5	LinBit decreased the number of fetuses with neural tube defects, decreased FPG, and increased matermal circulating insulin	(45)
C57BL/6J mice, 100 ppb As^III^ (dw) for 24 weeks	Folate, 10 mg/kg of food supplement for 24 weeks	High folate supplementation improved iAs-induced insulin resistance and stimulated iAs metabolism in females.	(75)
Wistar rats, 5 mg/kg/day (og), for 30 days	Curcumin, 15 mg/kg/day (og), 30 days	Curcumin supplementation prevented iAs-induced changes in serum markers of hepatic and renal function.	(65)

Although oxidative damage is a common observation following iAs exposure in many different tissues, the implications for ROS accumulation in β-cells may be unique. For instance, a recent meta-analysis of iAs-exposure studies in mice and rats indicated that iAs tends to decrease expression levels of key antioxidant genes (92). These include superoxide dismutase, catalase, and glutathione-peroxidase, among others. This is inverted compared to the response to iAs in β-cells, which manifests as increased expression of antioxidant genes, presumably to limit changes to the cellular redox state (27). This may be because glucose-induced insulin secretion in β-cells is partly mediated by relatively small changes in redox status (26). These cells are so sensitive to ROS that incubation with just 1 μM H_2_O_2_ affects basal insulin secretion (26), and just 100 μM H_2_O_2_ significantly decreases viability (98). In stark contrast, 250 μM H_2_O_2_ is used as a moderate positive control to quantify accumulation of ROS in liver cell lines (99). Thus, this unique sensitivity highlights the need to study the effects of iAs on β-cells directly, and not to rely too heavily on studies in other tissues.

## Cytotoxicity, Autophagy, and the Cell Cycle

Indications of cytotoxicity, disrupted autophagy, or apoptosis have been observed in β-cell lines using concentrations of iAs as low as 1 μM, although the minimum threshold for toxic effects vary with cell line and duration of exposure (46, 70, 84). Reduced viability as measured by reducing potential has been observed in the MIN6 cell line following 24 h exposure to ≥1 μM arsenite (although reducing potential may also be affected by changes to cellular energetics independent of toxicity), with a 50% reduction in viability at approximately 5 μM and activation of the antioxidant-response gene expression program (79). The INS-1 line exposed to iAs for just 24 h showed significantly decreased proliferation at ≥2.5 μM, with decreased mitochondrial membrane potential and increased cytoplasmic cytochrome c, indicative of autophagy (85). This cell line at 1–2 μM iAs exposure also exhibits reduced oxygen consumption capacity and viability (90). Pan et al estimated the IC50 for INS-1 cells to be about 30 μM (85). By comparison, isolated islets exposed to 20–50 μM iAs for 24 h exhibited >50% islet destruction, suggesting that islets, INS-1 cells, and MIN6 cells may be similarly sensitive to the cytotoxic effects of iAs (100).

There is substantial evidence that chronic *in vivo* iAs exposure disrupts autophagy in other tissues. In one such study, 20 weeks of exposure to 50 ppm iAs in drinking water during high-fat diet administration significantly induced hepatic expression of 17 out of 21 autophagy-related genes examined, with the remaining 4 genes trending toward an increase. This was also accompanied by a significant increase in hepatic lipid peroxidation and ROS accumulation (76). In β-cell lines as well, investigators have observed iAs-induced changes in autophagy (51, 56, 84), often noting enhanced levels of the autophagy marker LC3-II.

There is some debate about whether autophagy induced by iAs in β-cells is mediated by ROS. Some investigators have found that iAs induces autophagy in an ROS-dependent fashion (84). Other groups using non-β-cell lines have shown that autophagy can be activated in the absence of excessive ROS generation, and have therefore concluded that iAs-induced autophagy is ROS-independent (99). That autophagy induction occurs at comparable doses of iAs in other tissues without significant ROS accumulation suggests that perhaps β-cells, while susceptible to ROS, may also be affected by parallel, ROS-independent iAs-induced autophagy. This may be considered an unresolved topic in the field and additional mechanistic investigations at environmentally-relevant concentrations of iAs are warranted.

## Serotonin Metabolism

In mouse β-cells and islets, and to a lesser extent in human islets, serotonin regulates glucose-induced insulin secretion and proliferation (101, 102). Several parameters determine the concentration and effects of serotonin in β-cells, including serotonin production, serotonin disposal, and the specific distribution of serotonin receptors (103, 104). IAs exposure was recently observed to enhance serotonin disposal in the MIN6-K8 line by upregulation of the serotonin disposal gene *Ugt1a6a*. The upregulation phenomenon in response to iAs exposure was replicated in mouse islets, and the same pattern was observed for the human homolog of this gene, *UGT1A6*, in human islets upon chronic exposure to iAs (23). It is not clear what pathways are responsible for induction of *Ugt1a6a*, however *Ugt1a6a* expression is known in other tissues to be regulated by Nrf2 and the aryl hydrocarbon receptor. As *Ugt1a6a* was previously unappreciated as a regulator of β-cell function, this study highlights how EDCs such as iAs can be utilized to identify novel regulators of glucose-induced insulin secretion. Further study is warranted to evaluate the translatability of this work to animal models and cases of human exposure.

## Considerations and Future Directions

**Figure 1:**
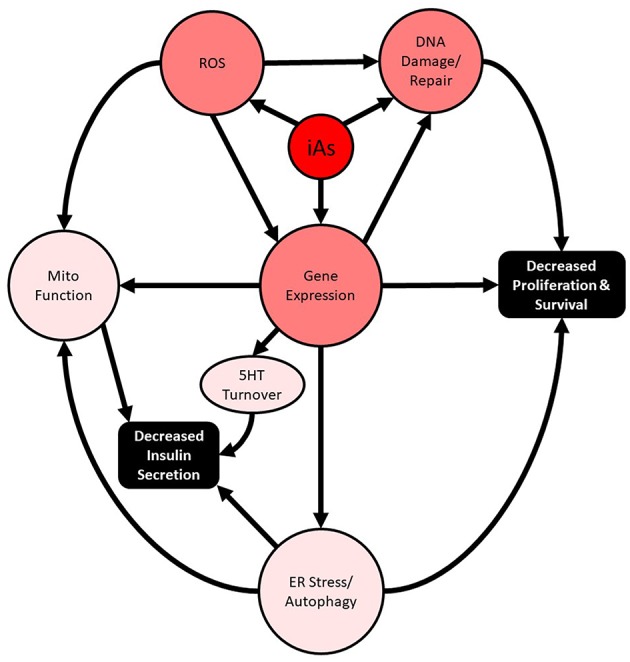
Summary of routes to iAs-induced toxicity and insulin-secretory effects.

Importantly, these studies intersect two major public health crises: chronic arsenic exposure and diabetes. In the past 20 years, substantial supporting evidence for the involvement of disrupted autophagy and oxidative damage as the major mediators of iAs-induced pancreatic β-cell dysfunction, manifesting as altered cell survival and impaired insulin secretion, has been reported both *in vivo* and *in vitro* (Figure 1). In more recent years, the concentrations used to study the phenomenon have decreased dramatically, and the lengths of exposure time have increased. These are positive trends for the field and are largely possible as a result of more sensitive analytical methods that are now more widespread. The use of these techniques has enabled the relatively recent discovery that MMA and DMA have different activities in β-cells and should be further explored. Now that human pancreatic islets are more widely available for research purposes throughout the world, replication of animal model findings in human islets is a more practical and reasonable option.

With the largest population-scale exposures of arsenic ongoing in developing or impoverished nations, it is less likely that synthetic therapeutic interventions targeting β-cells (without broader applicability to arsenic-independent β-cell function) may find traction at the levels of commercial development and clinical use. Appropriately, interventional studies aimed at improving β-cell resistance to arsenic exposure appear designed in consideration of this fact, mostly utilizing relatively inexpensive nutritional supplementation that may also have more systemic benefits to arsenic-exposed individuals (Table 3). Thus, the model system research presented here provide evidence that optimal nutrition rich in natural antioxidants may improve β-cells' resistance to chronic arsenic exposure *in vivo*.

The problem of arsenic exposure through contaminated drinking water is ultimately addressable at the level of public policy. Though diabetes itself may feel less immediate than acutely-life-threatening afflictions associated with arsenic exposure (such as cancer, cardiovascular disease, and nephropathy), highlighting the diabetes link provides yet another mechanism by which the scientific community can provide lawmakers and policy officials with justification to prioritize access to clean and safe water. That population-level arsenic exposure has been a known problem for more than 30 years in Bangladesh alone, with other pockets of exposure around the globe, reveals a global failure of institutions to address the needs of the impoverished and exposed. As water treatment technology and infrastructure are developed to address the pressing dangers of arsenic exposure, they will undoubtedly reduce economic and inertial barriers to the further improvement of water quality. Future studies and research communications might emphasize the preventability of chronic arsenic exposure as a point of discussion in the hopes that the issue can be continually brought to the forefront of public concern.

## Author Contributions

CC and SS conceived, authored, revised, and approved the manuscript and take responsibility for its publication.

### Conflict of Interest Statement

The authors declare that the research was conducted in the absence of any commercial or financial relationships that could be construed as a potential conflict of interest.
